# Moving Beyond Outcome-Based Exercise Studies in Cognitive Aging: A Mechanism-Informed Narrative Review of Cerebrovascular Anchors and Supportive Biological Endpoints

**DOI:** 10.3390/life16081283

**Published:** 2026-08-03

**Authors:** Zhigang Zhao, Dong Wang, Wei Gao, Chun-Hsien Su

**Affiliations:** 1 Physical Education Teaching and Training Department, Shanxi Institute of Technology, Yangquan 045000, China; zhaozhigang@sxit.edu.cn; 2 Sports Rehabilitation Department of Sports Teaching Department, Shanxi Medical University, Jinzhong 030604, China; wangdong@sxmu.edu.cn; 3 Graduate Institute of Sports Training, University of Taipei, Taipei 111036, Taiwan; 4 Department of Physical Education, Putian University, Putian 351100, China; 5 Department of Exercise and Health Promotion, Chinese Culture University, Taipei 111369, Taiwan

**Keywords:** vascular cognitive vulnerability, biological responsiveness, neurovascular coupling, exercise dose, mechanistic endpoints, responder heterogeneity

## Abstract

Exercise training is frequently examined in relation to cognitive and brain health in aging populations. Yet many exercise–cognition studies remain difficult to interpret mechanistically because cognitive outcomes are often assessed without sufficient alignment with exercise dose, biological endpoints, or participant vulnerability. This mechanism-informed narrative review argues that future research in cognitive aging should move beyond outcome-based interpretation alone and more explicitly connect the exercise stimulus with cerebrovascular endpoints, supportive biological markers, baseline vulnerability, and cognitive or translational outcomes. The review treats cerebrovascular endpoints, including cerebral blood flow, cerebrovascular reactivity, endothelial function, vascular compliance, and neurovascular coupling, as primary mechanistic anchors because they are closely related to vascular cognitive vulnerability and are more proximal to biological adaptation than cognitive test scores alone. Immune–inflammatory markers, blood–brain barrier-related indicators, myokines, neurotrophic factors, and exercise-induced circulating factors are considered supportive endpoints because they may clarify broader biological responsiveness when selected according to a clear hypothesis. The review also considers how exercise modality, delivered dose, adherence, baseline fitness, vascular risk, cognitive status, sex, comorbidity, and recovery capacity may shape responder heterogeneity. The purpose of this review is to develop a mechanism-informed framework for interpreting exercise–cognition studies in aging by aligning the delivered exercise stimulus, participant vulnerability, biological responsiveness, and cognitive or translational outcomes. Based on this synthesis, we propose a five-layer endpoint framework that positions cerebrovascular measures as primary mechanistic anchors and supportive biological markers as hypothesis-dependent contextual endpoints. Its distinct contribution is to integrate these elements within a single interpretive structure rather than considering exercise exposure, biological responses, participant characteristics, and cognitive outcomes as separate domains. Rather than serving as a clinical prescription model or formal reporting guideline, the framework is intended to improve hypothesis–endpoint alignment and the mechanistic interpretation of exercise–cognition studies in aging.

## 1. Introduction

Cognitive aging is commonly assessed through changes in memory, executive function, attention, processing speed, and global cognitive performance. These outcomes are clinically meaningful, but they sit relatively far downstream from the biological processes that exercise interventions are assumed to modify. Age-related cognitive vulnerability is shaped by interacting vascular, metabolic, inflammatory, and neurobiological factors, including cerebrovascular regulation, endothelial function, neurovascular coupling, cardiometabolic burden, and systemic inflammatory status [1,2,3]. This is where many exercise–cognition studies become difficult to interpret mechanistically. A change in cognitive test performance may reflect neural, vascular, or systemic adaptation. Yet the same change can also be shaped by baseline risk, outcome selection, learning effects, medication use, comorbidities, adherence, or insufficient exposure to the intended exercise stimulus [4]. Conversely, the absence of measurable cognitive improvement should not be read too quickly as evidence that exercise produced no biological adaptation. Vascular, inflammatory, fitness-related, or brain structural changes may occur before cognitive changes become detectable, may affect only selected cognitive domains, or may require longer follow-up before they become functionally meaningful.

Exercise training has been widely investigated as a strategy to support cognitive and brain health in middle-aged and older adults. A systematic review and meta-analysis suggests that exercise interventions may improve selected cognitive domains, including executive function and memory, although the observed effects vary across populations, modalities, intervention duration, adherence, and cognitive outcome measures [5]. Human experimental evidence also supports the biological plausibility of exercise-related brain adaptation, including findings that aerobic exercise training can influence hippocampal volume and memory in older adults [6]. Broader reviews of physical activity, cognition, and brain outcomes further suggest that exercise may influence brain health through several interacting pathways rather than through a single cognitive or neural mechanism [7]. Aerobic exercise has received particular attention because of its links with cardiorespiratory fitness, vascular regulation, and cerebrovascular responsiveness, whereas resistance, multimodal, and mind–body exercise are increasingly studied but are less consistently integrated with mechanistic endpoints [5,8,9,10]. This heterogeneity may reflect more than inconsistency across studies; it may indicate that exercise–cognition effects depend on whether an intervention delivers an adequate biological stimulus for a given participant profile and whether the study captures the intermediate mechanisms most likely to connect exercise exposure with cognitive outcomes.

Existing reviews have mapped important but largely distinct components of this field, including exercise effects on cognitive outcomes, cerebrovascular function, vascular aging, blood–brain barrier-related mechanisms, and muscle–brain signaling [5,8,11,12,13,14]. Collectively, this literature supports the biological plausibility of exercise as a strategy for brain health in aging, but it generally addresses individual outcome domains or mechanistic pathways rather than their alignment within the same exercise study. Cognitive-outcome reviews may establish whether exercise is associated with cognitive change without clarifying whether the delivered stimulus engaged the expected biological pathway [5]; cerebrovascular reviews may characterize vascular adaptation without fully integrating participant vulnerability, delivered exercise dose, or supportive biological responses [8,11]; and reviews of blood–brain barrier-related mechanisms [13] or muscle-derived signaling [12,14] often address mechanisms whose human relevance varies across populations and evidence levels. The distinct contribution of the present review is therefore to move from domain-specific synthesis toward mechanism-informed endpoint alignment. Rather than proposing another catalog of biomarkers or another summary of exercise efficacy, we integrate participant vulnerability, the exercise stimulus, primary cerebrovascular endpoints, supportive biological endpoints, and cognitive or translational outcomes within a single interpretive structure.

The emphasis on cerebrovascular biology in this framework should not be interpreted as implying that vascular pathways alone determine cognitive aging or exercise-related cognitive adaptation. Complementary influences include sleep and psychological well-being, endocrine and metabolic health, and structural and functional brain plasticity, which may interact with vascular regulation, systemic biological responses, and participant vulnerability. Structural and functional brain plasticity also represents an important complementary pathway through which physical activity and exercise may influence brain and cognitive health [7]. These complementary pathways are acknowledged within the broader biological context of the framework but are not reviewed comprehensively here, because the present review intentionally prioritizes cerebrovascular endpoints as mechanistic anchors and selected immune–inflammatory, blood–brain barrier-related, muscle-derived, and neurotrophic signals as supportive endpoints.

**Figure 1:**
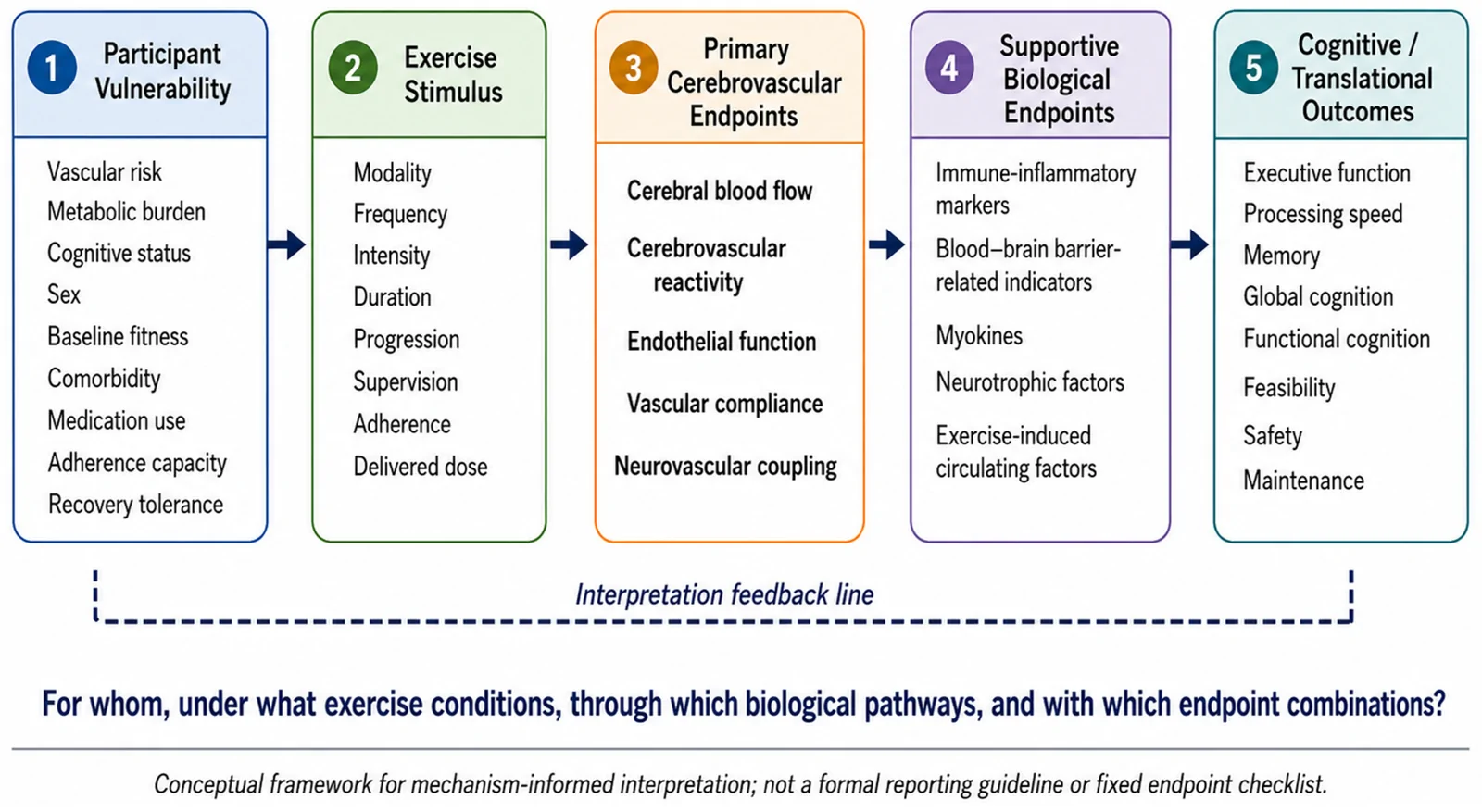
Mechanism-informed endpoint framework for exercise studies in cognitive aging. The framework links five interpretive layers: participant vulnerability, exercise stimulus, primary cerebrovascular endpoints, supportive biological endpoints, and cognitive or translational outcomes. Cerebrovascular measures are positioned as primary mechanistic anchors, whereas immune–inflammatory, blood–brain barrier-related, muscle-derived, and neurotrophic markers provide hypothesis-driven supportive context. Cognitive and translational outcomes are interpreted in relation to the preceding layers rather than as isolated endpoints. The dashed feedback line indicates that observed outcomes may inform subsequent interpretation and refinement of participant characterization, exercise exposure, or endpoint selection; it does not imply a bidirectional causal pathway. The framework is conceptual and is not intended as a formal reporting guideline or fixed endpoint checklist.

Accordingly, the purpose of this review is to develop a mechanism-informed framework for interpreting exercise–cognition studies in aging by explicitly aligning participant vulnerability, the delivered exercise stimulus, biological responsiveness, and cognitive or translational outcomes. Cerebrovascular endpoints—including cerebral blood flow, cerebrovascular reactivity, endothelial function, vascular compliance, and neurovascular coupling—are positioned as primary mechanistic anchors because of their relevance to vascular cognitive vulnerability, whereas immune–inflammatory markers, blood–brain barrier-related indicators, myokines, and neurotrophic factors are treated as supportive endpoints whose interpretation depends on the specific hypothesis and strength of the available evidence. Exercise modality, frequency, intensity, duration, progression, adherence, and delivered dose are incorporated as characteristics of the biological stimulus rather than as reporting details alone [15,16,17], while participant vulnerability and responder heterogeneity are considered with appropriate biological and statistical caution [18,19]. The new perspective offered by the framework lies in integrating these elements as linked interpretive layers rather than separate evidence domains, thereby enabling future studies to ask not only whether exercise influences cognition, but also for whom, under what exercise conditions, and through which measurable biological pathways such responses may emerge. Figure 1 summarizes this five-layer endpoint-alignment framework.

## 2. Narrative Search Strategy and Conceptual Synthesis Approach

This article takes the form of a mechanism-informed narrative review rather than a systematic review, scoping review, or meta-analysis. The central question was therefore not how large the pooled effect of exercise on cognition might be, but how evidence from exercise training, cerebrovascular function, biological markers, and participant vulnerability can be brought together to improve the interpretation of exercise–cognition studies in aging. This positioning follows the broader distinction among review types [20,21]. Narrative and conceptual reviews are useful when the task is to interpret heterogeneous evidence and develop an integrative argument, whereas systematic reviews and meta-analyses are designed for explicit eligibility-based evidence identification, appraisal, and effect estimation. Accordingly, the review focused on the literature related to exercise training, cognitive aging, cerebrovascular regulation, immune–inflammatory and muscle-derived signaling, exercise-dose characteristics, intervention reporting, and responder heterogeneity.

A structured narrative search was conducted using PubMed, Web of Science, Scopus, and Google Scholar. Rather than relying on a single fixed search string, the search was organized around several concept clusters. These clusters covered exercise exposure terms, including exercise training, physical activity, aerobic exercise, resistance training, multimodal exercise, and mind–body exercise; population and cognition terms, including cognitive aging, subjective cognitive decline, mild cognitive impairment, and vascular cognitive impairment; mechanistic endpoint terms, including cerebral blood flow, cerebrovascular reactivity, endothelial function, neurovascular coupling, blood–brain barrier, inflammation, cytokines, myokines, and brain-derived neurotrophic factor; and interpretation-related terms, including exercise dose, adherence, intervention reporting, and individual response. Sources were prioritized for inclusion when they directly informed at least one component of the proposed framework: (1) exercise exposure or intervention characteristics; (2) cognitive aging or cognitive outcomes in middle-aged, older, or cognitively at-risk adults; (3) cerebrovascular regulation or related vascular endpoints; (4) supportive immune–inflammatory, blood–brain barrier-related, muscle-derived, or neurotrophic pathways; or (5) participant vulnerability, intervention reporting, adherence, or interindividual response. Priority was given to human intervention studies, systematic reviews, meta-analyses, and other evidence syntheses directly relevant to exercise–cognition or mechanistic endpoint interpretation. Methodological papers, reporting frameworks, and mechanistic reviews were included when they informed measurement, intervention description, or interpretation of the proposed framework. Selected translational or preclinical studies were retained when they provided mechanistic context for candidate pathways that were not yet established directly in humans. Studies without clear relevance to cognitive aging, exercise exposure, or the mechanistic and interpretive domains of the framework were not prioritized. Because cognitive outcomes in non-pharmacological interventions are heterogeneous, studies were considered particularly informative when they clarified the relationship between cognitive endpoints and biological or intervention-level measures [4].

Conceptual relevance, rather than exhaustive inclusion, guided the synthesis. Evidence was organized around five interpretive layers: participant vulnerability, exercise stimulus, primary cerebrovascular endpoints, supportive immune–inflammatory and muscle-derived endpoints, and cognitive or translational outcomes. This choice reflects the purpose of the review: to integrate evidence across heterogeneous domains and develop an interpretive framework, rather than to provide a complete inventory of all potentially relevant studies [21]. Intervention description and reporting principles were treated as part of the interpretive framework because incomplete reporting of exercise content, progression, supervision, and adherence limits replication and weakens mechanistic interpretation [15,16]. Exercise-dose concepts were interpreted with reference to established exercise prescription principles, including frequency, intensity, time, type, and progression [17]. Responder heterogeneity was considered cautiously because apparent individual differences in training response may reflect both true biological variability and measurement or statistical factors [18]. Sources were prioritized when they directly informed the interpretation of exercise exposure, cerebrovascular or supportive biological endpoints, participant vulnerability, cognitive outcomes, or the proposed endpoint-alignment framework. In this sense, the search strategy supported a mechanism-informed synthesis of conceptually relevant evidence, rather than pooled effect estimation or formal certainty grading. The narrative search was last updated in June 2026. Because the search was conducted for narrative conceptual synthesis rather than systematic evidence identification, record-level counts of retrieved, screened, and excluded articles were not prospectively recorded. Accordingly, a retrospective record-level screening flow or PRISMA-style study-selection count was not generated. The final synthesis comprised 64 cited sources, including 9 key human intervention or trial-derived reports and 18 systematic, scoping, meta-analytic, or umbrella evidence syntheses. The remaining sources provided mechanistic, observational, methodological, reporting, guidance, or selected translational/preclinical context relevant to the development and interpretation of the framework. Because the purpose of this review was conceptual synthesis rather than pooled effect estimation or formal evidence grading, no formal risk-of-bias assessment or certainty-of-evidence rating was performed. The implications of this methodological choice, together with other limitations of the evidence base and the proposed framework, are discussed in Section 9.

## 3. Why Outcome-Based Exercise–Cognition Studies Are Difficult to Interpret

Cognitive outcomes remain necessary in exercise studies of aging because they indicate whether an intervention is associated with changes that matter for brain health and daily function. However, cognitive test performance is a distal endpoint, and its mechanistic meaning is often uncertain. Studies also vary in the cognitive domains they emphasize, including memory, executive function, processing speed, attention, and global cognition, and these domains may not respond to exercise in the same way or over the same time course [4,5]. Meta-analytic evidence suggests that exercise training may be associated with cognitive benefits in older adults, but the magnitude and pattern of effects depend on cognitive domain, participant characteristics, training modality, intervention duration, and analytic choices [22,23,24]. This domain-specific variability is not a minor methodological inconvenience. It affects whether a cognitive change can reasonably be interpreted as evidence of vascular, neural, metabolic, or systemic adaptation.

Cognitive outcomes are also influenced by factors that may be only indirectly related to the exercise mechanism being tested, including baseline cognitive status, education, test familiarity, sleep, mood, medication use, comorbidities, and assessment timing. Repeated testing adds a further challenge because practice effects may occur as participants become familiar with task demands, response formats, or testing procedures [25]. Trials using repeated cognitive assessments should therefore prespecify the primary cognitive domain and, where appropriate, use validated alternate forms, consistent administration procedures, and comparable retest intervals across groups and assessment points. Small improvements on repeatedly administered measures should be interpreted cautiously when learning effects cannot be separated from intervention-related change. Cognitive test selection should also follow the mechanistic hypothesis rather than rely primarily on global screening tools. When cerebrovascular function or vascular cognitive vulnerability is central to the study question, domain-specific measures of executive function and processing speed may provide greater mechanistic relevance, whereas memory or other domains may be prioritized when different biological pathways are hypothesized. No cognitive domain should, however, be regarded as uniquely specific to a single biological mechanism. A positive cognitive change does not by itself identify the biological pathway through which exercise acted. Conversely, a null cognitive result does not necessarily indicate that the intervention failed to induce vascular, metabolic, inflammatory, fitness-related, or brain structural adaptation.

Exercise exposure adds another layer of uncertainty because nominally similar interventions may differ substantially in modality, dose, progression, supervision, adherence, and participant tolerance. Incomplete intervention reporting therefore limits replication and makes it difficult to determine whether mixed cognitive findings reflect biological response or differences in the delivered exercise stimulus [15,16,17]. These dose-related considerations are examined in greater detail in Section 6.

Biological adaptation and cognitive change may also unfold on different time scales. Exercise may first affect cardiorespiratory fitness, blood pressure regulation, endothelial function, cerebrovascular responsiveness, systemic inflammatory status, or brain structural and functional plasticity before cognitive changes become detectable. Mechanistic and translational reviews of exercise-related brain plasticity suggest that exercise can influence brain health through multiple interacting pathways, but these pathways may not be captured by a single cognitive test or short-term cognitive endpoint [7,26,27]. In some populations, intermediate biological adaptations may be more sensitive to the intervention than global cognitive scores, especially when baseline cognition is relatively preserved or when follow-up is short. Human exercise studies that include cerebrovascular measures suggest that biological and cognitive outcomes may provide complementary information rather than interchangeable indicators of benefit [8,9,10]. This distinction matters because cognitive outcomes alone cannot determine whether an intervention was biologically inactive, insufficiently dosed, poorly matched to the participant profile, or simply not assessed with endpoints sensitive to the expected mechanism.

Participant heterogeneity adds a further source of uncertainty. Baseline vascular risk, cognitive status, fitness, comorbidity, medication use, adherence, and recovery capacity may influence both exercise tolerance and biological or cognitive response, while apparent interindividual differences may also reflect measurement and statistical factors [18,28]. Cognitive outcomes are therefore most informative when interpreted alongside exercise exposure, intermediate biological endpoints, and baseline vulnerability; these issues are examined more fully in Section 7.

## 4. Cerebrovascular Endpoints as Primary Mechanistic Anchors

These limitations make cerebrovascular endpoints especially important for exercise–cognition research. Cognitive aging is closely related to vascular cognitive vulnerability, including altered cerebral perfusion, endothelial dysfunction, impaired autoregulation, arterial stiffness, and disrupted neurovascular coupling [1,2,3]. These processes are more proximal to brain vascular health than cognitive test scores alone and may therefore offer a more mechanism-relevant indication of exercise-related adaptation. Cerebral blood flow is regulated through interacting mechanisms involving perfusion pressure, arterial carbon dioxide, oxygen delivery, metabolic demand, autonomic regulation, and vascular structure [29]. In aging adults, cardiovascular and vascular changes can alter cerebral blood flow regulation and may contribute to cognitive vulnerability, while aerobic fitness and cardiovascular function may influence the preservation of cerebral perfusion [30,31]. In this context, cerebrovascular endpoints are not merely secondary biomarkers. They can serve as primary mechanistic anchors because they help determine whether an exercise intervention has engaged vascular pathways relevant to cognitive resilience [8].

Exercise training is likely to engage cerebrovascular function through more than one physiological route. Aerobic exercise, in particular, repeatedly increases cardiorespiratory and hemodynamic demand, exposing the vascular system to stimuli such as shear stress, autonomic adjustment, blood pressure regulation, and metabolic challenge. These stimuli may contribute to vascular adaptation when the intervention is sufficiently intense, progressive, and sustained. Reviews of exercise, cerebrovascular function, and cognition support the biological plausibility of exercise-related cerebrovascular adaptation in aging, although the magnitude and consistency of effects differ across populations, intervention designs, and measurement approaches [8]. Human intervention and training-status studies further suggest that aerobic exercise may influence selected aspects of cerebrovascular regulation and cognition in older adults, but these findings should be interpreted in relation to baseline risk, training adherence, and endpoint selection [9,10,32]. Exercise training has also been associated with changes in resting cerebral blood flow in older adults with and without cognitive impairment, and meta-analytic evidence suggests that physical exercise may improve cerebral blood velocity in older people [33,34]. However, these findings should not be read as evidence of a simple linear pathway from exercise to cerebrovascular improvement to cognitive benefit. They instead suggest that cerebrovascular endpoints can help clarify whether a given exercise stimulus is biologically active in a pathway relevant to cognitive aging.

Not all cerebrovascular endpoints answer the same mechanistic question, and their selection should depend on the pathway being tested. Cerebral blood flow provides information about perfusion, whereas cerebrovascular reactivity reflects the capacity of cerebral vessels to respond to vasoactive stimuli. Neurovascular coupling refers to the coordination between neural activity and local vascular response, and endothelial function reflects vascular health that may influence both peripheral and cerebral regulation [8,29,35,36,37]. Arterial stiffness and vascular compliance may also be relevant because large-vessel aging can affect pulsatile flow transmission and downstream microvascular stress, and arterial stiffness has been associated with cognitive outcomes in adult and older populations [30,38]. These endpoints do not measure the same vascular process and should not be interpreted as interchangeable. For example, a change in resting cerebral blood flow does not necessarily indicate improved cerebrovascular reactivity, and improved peripheral endothelial function does not automatically demonstrate improved brain vascular regulation.

The interpretation of cerebrovascular measures also depends strongly on how they are assessed. Cerebrovascular reactivity can be measured using approaches such as carbon dioxide challenge with MRI or other modalities, but findings may depend on the stimulus, imaging sequence, gas-delivery method, analytic approach, and physiological context [35,36]. Neurovascular coupling measures require similar caution because the observed signal reflects interactions among neural activity, vascular responsiveness, metabolic demand, and methodological assumptions [37]. Similarly, BOLD fMRI responses in aging may be affected by vascular as well as neural factors, which complicates interpretation when vascular function differs across age or risk groups [39]. Transcranial Doppler, near-infrared spectroscopy, MRI-based perfusion methods, peripheral vascular measures, and cardiometabolic indicators may each provide useful information, but they do not represent the same biological construct. This caution matters for the proposed framework: cerebrovascular endpoints strengthen mechanistic interpretation only when their physiological meaning and measurement limits are explicitly recognized.

Cerebrovascular endpoints therefore provide a necessary but not sufficient mechanistic layer for exercise–cognition research. They are necessary because they connect exercise exposure to biological processes directly relevant to vascular cognitive vulnerability. They are not sufficient because cognitive resilience is also shaped by systemic inflammation, blood–brain barrier-related mechanisms, muscle-derived signaling, baseline risk, adherence, and recovery capacity. For this reason, study designs are more informative when they align exercise dose and modality with selected cerebrovascular outcomes while also considering supportive biological markers and participant vulnerability. This approach keeps cerebrovascular function at the center of mechanistic interpretation without reducing cognitive aging to a single vascular pathway.

## 5. Immune–Inflammatory and Muscle-Derived Signals as Supportive Mechanistic Endpoints

Cerebrovascular endpoints provide the primary mechanistic anchors for this review, but they are unlikely to explain exercise-related cognitive resilience on their own. Cognitive aging is also shaped by systemic inflammation, blood–brain barrier-related mechanisms, metabolic regulation, skeletal muscle adaptation, and neurotrophic signaling. These processes may influence how an older adult responds to exercise and whether vascular adaptation is accompanied by cognitive or functional change. For this reason, immune–inflammatory and muscle-derived signals are best interpreted as supportive mechanistic endpoints [12,13,14,40]. Importantly, the strength and source of evidence differ substantially across these pathways. In the discussion below, findings from human exercise interventions and human observational studies are distinguished from mechanistic evidence derived primarily from translational or preclinical models. The presence of a plausible biological pathway, particularly when supported mainly by experimental models, should not be interpreted as evidence that the same pathway mediates cognitive effects of exercise in aging humans.

Systemic inflammatory markers and blood–brain barrier-related mechanisms are relevant because vascular cognitive vulnerability is not solely a hemodynamic problem. Low-grade inflammation, endothelial dysfunction, and altered barrier regulation may interact with cerebrovascular health and cognitive decline. Human observational and intervention evidence indicates that physical activity and exercise are associated with changes in peripheral inflammatory and neurotrophic profiles in older adults, although responses vary by marker, modality, population, and intervention duration [40,41,42]. Thus, peripheral inflammatory biomarkers represent one of the supportive domains with direct human exercise evidence. However, a change in a circulating inflammatory marker demonstrates systemic biological responsiveness rather than direct modification of central inflammation or neuroimmune signaling.

Blood–brain barrier-related mechanisms are most relevant when the research question concerns vascular integrity, inflammatory regulation, or neurovascular vulnerability. Exercise may influence blood–brain barrier physiology through pathways involving endothelial function, oxidative stress, inflammatory regulation, and vascular integrity [13]. However, compared with peripheral inflammatory responses, direct human exercise evidence for blood–brain barrier modification is less established, and much of the mechanistic interpretation remains translational, heterogeneous, or derived from disease-specific contexts. Reviews focused on Alzheimer’s disease and related neurodegenerative contexts support the biological plausibility that exercise may interact with neuroinflammatory pathways, but this evidence should be used cautiously when discussing general cognitive aging [43]. For the present framework, blood–brain barrier-related markers are most informative when they are selected to test a specific vulnerability profile or pathway, rather than when they are treated as broad indicators of brain health.

Muscle-derived and neurotrophic signals also broaden the interpretation of exercise-related biological responsiveness. Skeletal muscle is increasingly understood as an endocrine and paracrine organ that can communicate with other tissues through myokines and related exercise-responsive factors [12]. Candidate pathways relevant to brain health include brain-derived neurotrophic factor, FNDC5/irisin-related signaling, cathepsin B, and other factors that may connect muscle contraction, systemic metabolism, neuroplasticity, and cognition [14,44,45,46]. The evidence base, however, is not uniform across these candidates. Human exercise studies support measurable peripheral responses for BDNF and selected exercise-responsive factors, and cathepsin B has been examined in relation to exercise and memory in humans, whereas several proposed muscle-to-brain signaling mechanisms remain substantially informed by animal and translational work [14,44,45,46]. Even when a circulating factor is measurable in humans, this does not establish that peripheral concentrations reflect central nervous system activity or causally mediate cognitive improvement. Myokine and neurotrophic markers should therefore be interpreted as supportive endpoints, with the strength of mechanistic inference matched to the underlying evidence level.

Exercise-induced circulating factors have also drawn attention to systemic-to-brain signaling. For candidates such as GPLD1 and clusterin, the mechanistic evidence cited here is predominantly preclinical and translational, including plasma-transfer and experimental studies in aged models [47,48]. These studies suggest that exercise-related circulating factors may influence neurogenesis, memory, or brain inflammatory profiles and therefore provide important mechanistic hypotheses. However, they do not establish that GPLD1, clusterin, or related circulating factors mediate cognitive benefits of exercise in humans. Within the present framework, these candidates should therefore be regarded primarily as hypothesis-generating supportive pathways until their relevance, temporal response, and association with cognitive adaptation are established more directly in human exercise studies.

The implication is not that future exercise–cognition studies should measure every available inflammatory marker, myokine, or neurotrophic factor. Excessive biomarker collection without a clear mechanistic hypothesis may create additional interpretive complexity. Instead, supportive endpoints should be selected according to the expected biological response of the intervention and the vulnerability profile of the population. For example, inflammatory or blood–brain barrier-related markers may be particularly informative in participants with vascular or metabolic risk, whereas neurotrophic or muscle-derived markers may be more relevant when the intervention is expected to produce substantial neuromuscular or cardiorespiratory adaptation. These pathways should therefore be interpreted as supportive and hypothesis-generating mechanisms rather than established causal mediators of cognitive improvement. Their relevance depends strongly on the exercise stimulus being tested, which makes exercise dose, modality, adherence, and progression central to the next level of interpretation. Table 1 summarizes the main cerebrovascular and supportive biological endpoint domains considered in this review.

## 6. Exercise Dose, Modality, and Biological Responsiveness

Exercise dose and modality are not simply descriptors of an intervention; they determine what biological stimulus is actually being tested. In mechanism-informed exercise–cognition research, frequency, intensity, duration, type, progression, supervision, adherence, and delivered dose shape whether the intended stimulus is strong enough, tolerable enough, and sustained enough to generate vascular, metabolic, neuromuscular, inflammatory, or neurotrophic responses. Dose–response evidence in older adults suggests that exercise effects on cognition may depend on cognitive status, session duration, frequency, total exposure, and the way dose is quantified, although dose parameters do not consistently predict cognitive effects across populations [49]. Incomplete reporting of intervention features limits replication and makes it difficult to interpret whether null or mixed cognitive findings reflect an ineffective intervention, insufficient stimulus, poor adherence, or endpoint mismatch [15,16]. Established exercise prescription principles provide a useful language for describing dose, but this review is not primarily concerned with prescription guidance. Rather, exercise characteristics should be reported and interpreted in relation to the biological pathway that the study aims to engage [17].

Aerobic exercise has received the most attention in this literature because it maps readily onto cardiorespiratory fitness, cerebrovascular responsiveness, and cognitive outcomes. The emphasis is understandable from a mechanistic perspective because aerobic training repeatedly exposes the cardiovascular and cerebrovascular systems to hemodynamic demand, shear stress, autonomic adjustment, and metabolic challenge. These stimuli may support endothelial and cerebrovascular adaptation, particularly when the intervention is sufficiently intense, progressive, and sustained [8]. Human intervention studies suggest that aerobic exercise may influence selected cerebrovascular and cognitive outcomes in older adults, although responses vary according to baseline risk, adherence, and measurement approach [9,10]. Observational or training-status comparisons also suggest that regular aerobic training may be associated with more favorable cerebrovascular and cognitive profiles [32]. Broader meta-analytic evidence indicates that exercise interventions, including aerobic and resistance-based approaches, may improve cognitive function in older adults, but such findings should be interpreted with attention to modality, dose, baseline status, and outcome selection [50]. Aerobic exercise should therefore not be assumed to be the appropriate stimulus for all exercise–cognition questions. Its relevance depends on whether the study hypothesis emphasizes cardiorespiratory fitness, vascular regulation, perfusion-related endpoints, or cognitive domains expected to be sensitive to these pathways.

Resistance exercise brings a different stimulus profile into exercise–cognition research. Resistance training places greater emphasis on neuromuscular loading, muscle strength, insulin sensitivity, functional capacity, and muscle-derived signaling. Systematic review evidence suggests that resistance training may improve cognitive function in older adults with different cognitive status, while randomized trial evidence indicates that resistance training can benefit selected executive functions in older women [51,52]. These findings justify including resistance training in mechanism-informed exercise–cognition research, but they should not be interpreted as evidence that resistance exercise is superior to other modalities. For the present framework, resistance training is important because it may provide a neuromuscular and metabolic stimulus that differs from the hemodynamic emphasis of aerobic exercise. Its mechanistic relevance depends on whether the study aims to test strength-related, metabolic, muscle-derived, functional, or cognitive pathways.

Multicomponent exercise can combine aerobic, resistance, balance, coordination, or flexibility components and may therefore deliver a broader intervention stimulus. That breadth can be useful, but it also creates interpretive challenges. Evidence from older adults with cognitive impairment suggests that multicomponent physical exercise may affect global cognition, particularly when aerobic elements are included, while broader evidence in older adults indicates potential effects on overall cognition and frailty-related outcomes [53,54]. These interventions may be especially relevant when functional capacity, balance, mobility, adherence, and cognitive engagement are part of the study hypothesis. However, when several components are delivered together, it may be difficult to determine which component, or which combination of components, is most closely related to observed cognitive or biological changes. Multicomponent interventions therefore require particularly careful reporting of component content, progression, adherence, and delivered dose.

Mind–body exercise and lower-intensity movement approaches raise a different interpretive issue. Modalities such as Tai Chi and Qigong may be relevant when the hypothesized mechanisms include balance, coordination, attentional engagement, autonomic regulation, stress reduction, social participation, or adherence. Meta-analytic and meta-regression evidence suggests that Tai Chi and Qigong may have beneficial effects on cognitive and physical functions in older adults, but these modalities should still be interpreted according to the biological and behavioral stimulus they deliver [55]. Their stimulus profile may differ substantially from interventions designed primarily to improve cardiorespiratory fitness or induce strong hemodynamic loading. If a study uses a lower-intensity or mind–body approach, the selected endpoints should correspond to the expected pathway rather than assume the same vascular or inflammatory responses expected from aerobic training.

The same logic applies to biomarker selection: biomarkers are most informative when their expected response matches the exercise stimulus being tested. Inflammatory, neurotrophic, and muscle-derived markers may be more meaningful when the intervention is expected to influence the biological system represented by those markers. Peripheral inflammatory and neurotrophic biomarkers in older adults may vary according to exercise modality, intervention duration, and participant characteristics [40]. Similarly, muscle-derived signals may be more relevant when the intervention produces substantial neuromuscular or metabolic adaptation [12,14]. Measuring these endpoints without considering the nature of the exercise stimulus can increase interpretive complexity rather than clarify mechanisms. Table 2 summarizes how major exercise modalities can be interpreted as distinct biological stimuli and how each modality may align with selected mechanistic endpoints. Exercise modality and dose should therefore be selected and reported according to the biological pathway being tested. Because the same prescribed intervention may not produce the same biological response across participants, participant vulnerability and responder heterogeneity become the next level of interpretation [18,28].

## 7. Population Vulnerability and Responder Heterogeneity

The same prescribed exercise dose and modality may not produce the same biological or cognitive response across older adults. This variation should not be dismissed as background noise or reduced to a simple distinction between responders and non-responders. Individual response to exercise training can reflect true biological variability, but it can also be influenced by measurement error, regression to the mean, adherence, baseline status, and the statistical approach used to classify response [18,28]. For exercise–cognition research, this caution matters because cognitive outcomes are often distal, variable, and sensitive to several non-exercise influences. A participant who shows little cognitive change may still have experienced meaningful vascular, metabolic, or fitness-related adaptation, whereas cognitive improvement in another participant may remain difficult to attribute to a specific biological pathway without aligned mechanistic endpoints. Responder heterogeneity is most useful when it is read in relation to exercise exposure, mechanistic endpoints, and baseline vulnerability.

Vascular and metabolic vulnerability are especially informative because they may affect both cerebrovascular reserve and exercise tolerance. Older adults with hypertension, obesity, insulin resistance, arterial stiffness, endothelial dysfunction, or vascular cognitive risk may differ in both cerebrovascular reserve and tolerance of a prescribed exercise dose. These factors may influence whether exercise produces sufficient hemodynamic or metabolic stimulus to alter cerebrovascular responsiveness, and they may also affect whether biological adaptation is accompanied by cognitive change [1,2,3]. Human exercise trials in vascular cognitive impairment and related at-risk populations suggest that baseline vascular burden and cardiovascular risk can shape the magnitude and interpretation of cognitive response to aerobic training [56,57]. In some individuals, greater vascular or metabolic risk may indicate more modifiable dysfunction; in others, the same risk profile may reflect more advanced vascular burden, reduced adaptive capacity, or greater difficulty sustaining the intended intervention. This makes baseline vulnerability part of the interpretation itself, rather than a peripheral clinical detail.

Baseline cognitive status also affects how exercise responses should be interpreted. Healthy older adults, individuals with subjective cognitive concerns, those with mild cognitive impairment, and adults with vascular cognitive vulnerability are unlikely to show the same outcome sensitivity, ceiling effects, disease burden, or time course of change. When baseline cognition is relatively preserved, global cognitive measures may be insensitive to early biological adaptation. When impairment is more pronounced, cognitive outcomes may be influenced by heterogeneous pathology, comorbidity, or reduced capacity to tolerate higher exercise loads. Exercise intervention evidence in older adults with mild cognitive impairment or subjective memory impairment supports the relevance of baseline cognitive status, but it also illustrates that cognitive response can vary by population, intervention design, and follow-up duration [58,59]. Participant selection and cognitive endpoint selection therefore need to be considered together. A study designed to test cerebrovascular responsiveness may require different cognitive endpoints from a study designed to examine functional capacity, neurotrophic signaling, or adherence in adults with greater cognitive vulnerability.

Other participant characteristics further complicate biological responsiveness, including sex, baseline fitness, comorbidity, medication use, recovery capacity, and adherence. Sex differences are particularly relevant because aging, vascular risk, hormonal history, and exercise adaptation may interact in ways that influence brain and cognitive outcomes [19]. Baseline fitness may determine whether a given intervention represents a sufficient challenge, an insufficient stimulus, or an excessive burden, while the delivered dose may differ from the prescribed dose when participants have frailty, fatigue, mobility limitations, low motivation, or competing health burdens [49]. Adherence is therefore not only a practical issue but also an interpretive issue. Reviews of exercise interventions in older adults with mild cognitive impairment or dementia, and broader reviews of exercise adherence in chronic conditions, suggest that adherence is shaped by individual, program-level, health-related, and behavioral factors [60,61]. Biomarker responses may also vary by modality, duration, and participant characteristics, which reinforces the need to interpret inflammatory, neurotrophic, and muscle-derived endpoints in relation to the population being studied [40].

**Figure 2:**
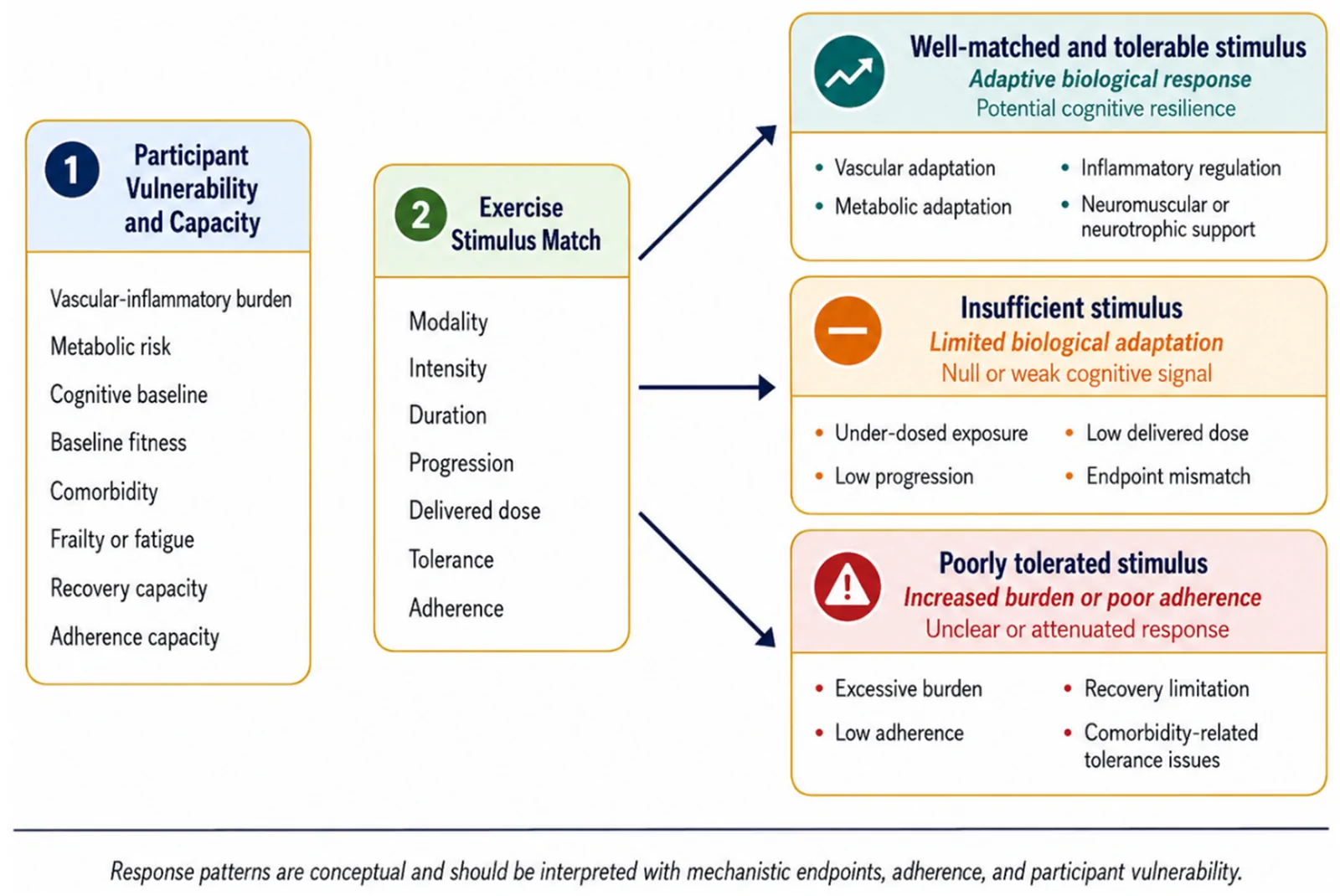
Conceptual response patterns linking participant vulnerability, exercise stimulus match, and biological responsiveness in exercise–cognition studies of aging. Participant vulnerability and capacity, including vascular-inflammatory burden, metabolic risk, cognitive baseline, baseline fitness, comorbidity, frailty or fatigue, recovery capacity, and adherence capacity, may influence whether a prescribed exercise stimulus is sufficiently matched, under-dosed, or poorly tolerated. A well-matched and tolerable stimulus may support adaptive biological responses, such as vascular and metabolic adaptation, inflammatory regulation, and neuromuscular or neurotrophic support, which may contribute to cognitive resilience. In contrast, an insufficient stimulus may produce limited biological adaptation and a weak or null cognitive signal, whereas a poorly tolerated stimulus may increase burden, reduce adherence, or produce unclear and attenuated responses. These response patterns are conceptual and should be interpreted in relation to mechanistic endpoints, delivered dose, adherence, recovery capacity, and baseline participant vulnerability rather than as fixed responder categories or clinical prescription rules.

Participant vulnerability should therefore be considered prospectively, rather than used only after the fact to explain inconsistent results. Its role is not to justify premature individualized exercise prescriptions, but to improve the design and interpretation of mechanism-informed studies. Baseline vascular risk, cognitive status, sex, fitness, comorbidity, adherence capacity, and recovery tolerance can help determine whether a given exercise stimulus is likely to be sufficient, tolerable, and biologically measurable. Figure 2 illustrates how exercise dose may interact with vascular-inflammatory vulnerability, fitness, adherence, and recovery capacity to shape adaptive, insufficient, or poorly tolerated biological responses. When vulnerability, exercise stimulus, and endpoint selection are aligned prospectively, exercise–cognition studies may be better positioned to explain who responds, under what conditions, and through which biological pathways. This logic brings the preceding sections together and leads to the mechanism-informed endpoint framework proposed for future exercise–cognition research.

## 8. A Mechanism-Informed Endpoint Framework for Future Exercise–Cognition Studies

The preceding sections suggest that exercise–cognition research in aging would benefit from stronger mechanistic alignment rather than simply adding more outcomes. Cognitive outcomes remain essential, but they are difficult to interpret when exercise exposure, biological responsiveness, and participant vulnerability are not evaluated together [4]. Similarly, mechanistic interpretation is limited when the prescribed and delivered exercise stimulus is not described with enough detail to support replication and biological interpretation [15,16]. Exercise–cognition studies can also be understood as complex interventions because their effects may depend not only on intervention content, but also on participant characteristics, implementation, adherence, context, and the pathways through which change is expected to occur [62,63,64]. The proposed framework therefore organizes interpretation around five linked layers: participant vulnerability, exercise stimulus, primary cerebrovascular endpoints, supportive biological endpoints, and cognitive or translational outcomes. As summarized in Figure 1, these layers are intended to be interpreted together rather than as separate domains.

Within this structure, participant vulnerability defines the context in which the delivered exercise stimulus is received [18,19,28], whereas modality, frequency, intensity, duration, progression, supervision, adherence, and delivered dose define the biological exposure being tested [15,16,17]. These two layers should be considered jointly because an exercise stimulus that is sufficient and recoverable for one participant profile may be insufficient, poorly tolerated, or biologically mismatched for another. The central design question is therefore not simply whether exercise was prescribed, but whether the delivered intervention was appropriate for the participant profile and capable of engaging the biological pathway being tested.

Cerebrovascular measures provide the primary mechanistic anchors and should be selected according to the specific vascular process of interest rather than treated as interchangeable markers [8,35,36,37]. Supportive immune–inflammatory, blood–brain barrier-related, myokine, and neurotrophic endpoints may add biological context when justified by the hypothesis, with the strength of mechanistic inference explicitly matched to whether the supporting evidence is human, translational, or preclinical [12,13,14,40]. Cognitive and translational outcomes should likewise be aligned with the expected mechanism and population [4], while accounting for adherence, safety, feasibility, tolerance, and maintenance. In this way, the five layers function as an integrated interpretive structure rather than as independent categories of measurement.

### 8.1. Practical Implications for Study Design and Endpoint Selection

In practical terms, the framework is intended to be applied prospectively, beginning with the biological question rather than with the availability of individual measures. Investigators should first specify the pathway expected to respond to the intervention and characterize the participant features most likely to influence exposure, tolerance, or responsiveness. The exercise intervention should then be described and monitored as a delivered biological stimulus, including modality, frequency, intensity, duration, progression, supervision, and adherence [15,16,17]. Endpoint selection should follow the mechanistic hypothesis: studies centered on vascular adaptation should prioritize the cerebrovascular measure most relevant to the process of interest, recognizing that cerebral blood flow, cerebrovascular reactivity, endothelial function, vascular compliance, and neurovascular coupling are not interchangeable [29,30,31,32,33,34,35,36,37,38,39]. Supportive immune–inflammatory, blood–brain barrier-related, myokine, or neurotrophic measures should be added when they address a prespecified secondary pathway rather than simply expanding the biomarker panel [13,40,41,42,43,44,45,46,47,48]. Cognitive outcomes should likewise be chosen according to the hypothesized mechanism, participant profile, and anticipated time course of change, with attention to practice effects and measurement sensitivity [4,22,23,24,25,58,59]. For vascular-centered hypotheses, domain-specific assessment of executive function and processing speed may be more informative than reliance on global cognitive screening alone, while validated alternate forms and consistent retest conditions should be used where feasible to reduce ambiguity from repeated testing [25].

Practical application of the framework does not require every study to measure every layer comprehensively. A pragmatic core measurement strategy for a mechanism-focused trial would include a well-characterized delivered exercise stimulus, including adherence and tolerance [15,16,17]; one primary cerebrovascular endpoint selected to match the hypothesized pathway; supportive biological markers only when they address a prespecified secondary mechanism; and a cognitive outcome selected for mechanistic relevance. The specific measurement approach should be proportional to the study question, population, available resources, participant burden, and expected timing of adaptation. In settings with limited access to advanced neuroimaging, transcranial Doppler-based cerebral blood-flow velocity or cerebrovascular reactivity, peripheral vascular measures, and targeted circulating biomarkers may provide feasible hypothesis-driven options, although these measures should not be interpreted as equivalent to direct regional perfusion or brain-specific vascular measures. Where resources permit, arterial spin labeling MRI can characterize cerebral perfusion, whereas MRI-based cerebrovascular reactivity can assess vascular responsiveness [29,30,31,32,33,34,35,36]. Task-based BOLD fMRI or related approaches may provide information relevant to neurovascular coupling, although interpretation requires consideration of both neural and vascular contributions to the observed signal [37,39].

Measurement timing should also be specified according to the biological question. In longitudinal training studies, baseline and post-intervention measurements should be obtained under comparable physiological conditions, with assessment timing standardized relative to the final exercise session and, where feasible, time of day. When the objective is to characterize chronic adaptation, immediate post-exercise effects should be distinguished from sustained training-related changes. Acute mechanistic studies should prespecify pre-exercise and post-exercise measurement windows, particularly for circulating biomarkers whose responses may vary with sampling time, assay procedures, exercise modality, and participant characteristics [40,44,45,46]. Across repeated assessments, the same measurement modality, physiological stimulus, acquisition protocol, and analytic approach should be maintained whenever possible, particularly for cerebrovascular reactivity and neurovascular coupling measures [35,36,37]. These suggestions represent feasibility-informed design principles rather than mandatory minimum standards; the priority remains mechanistic alignment and interpretability rather than the number or technological complexity of measurements collected [62,63,64]. Representative procedures, timing considerations, feasibility, and major interpretive cautions for selected measures are summarized in Table A1.

Analytic strategy should likewise reflect the layered structure of the framework. When biological and cognitive outcomes are measured repeatedly, mixed-effects models can be used to examine longitudinal trajectories while accounting for within-participant correlation and differences in the timing or magnitude of response. Prespecified moderator analyses can test whether baseline characteristics such as vascular risk, cognitive status, fitness, sex, or comorbidity modify the effect of the exercise intervention, preferably through interaction terms rather than post hoc responder classification [18,28]. When the hypothesis concerns a mechanistic pathway, mediation analysis should be based, where possible, on temporally ordered measurements in which intervention exposure precedes change in the candidate biological mediator and the mediator precedes or accompanies subsequent cognitive assessment. Longitudinal mediation models may therefore be particularly useful when repeated biological and cognitive measurements are available. However, statistical mediation should be interpreted as evidence consistent with a proposed pathway rather than proof of biological causality, especially when mediator and outcome measurements are concurrent or when residual confounding and measurement error cannot be excluded.

Table 3 further clarifies how the framework can guide interpretation without becoming a fixed endpoint checklist. The table links common design questions with corresponding interpretive issues, endpoint implications, and cautionary considerations. Its purpose is to help researchers align the exercise stimulus, participant vulnerability, mechanistic endpoints, and cognitive or translational outcomes before interpreting intervention effects, rather than to prescribe a uniform endpoint battery.

### 8.2. Translational and Clinical Relevance

From a translational perspective, the framework may also help contextualize exercise responses in clinical and applied settings, although its current role remains interpretive rather than prescriptive. Baseline vascular and metabolic vulnerability, cognitive status, fitness, comorbidity, medication use, tolerance, adherence, and recovery capacity may help explain why the same prescribed intervention is feasible and biologically responsive in some older adults but insufficient or poorly tolerated in others. In clinical practice, these considerations may support a more individualized interpretation of exercise response rather than reliance on cognitive change alone. However, the framework should not currently be used to define biomarker thresholds, classify individuals as clinical responders or non-responders, or prescribe specific exercise doses on the basis of cerebrovascular or circulating biomarkers. Such applications would require prospective validation of the framework and the establishment of clinically meaningful decision thresholds. Its immediate translational value is therefore to encourage more contextualized interpretation of exercise response and closer alignment among participant characteristics, exercise exposure, safety, feasibility, and outcomes.

Taken together, the framework is intended to improve hypothesis–endpoint alignment and mechanistic interpretation rather than prescribe a uniform endpoint battery or establish causal certainty. By linking exercise exposure, participant context, biological responsiveness, and cognitive outcomes, it provides a structured and testable basis for future study design and interpretation while maintaining appropriate boundaries for clinical translation.

## 9. Limitations and Priorities for Framework Validation

Several limitations should be considered when interpreting this review. First, although the literature search was structured across multiple databases and concept domains, the review was designed as a narrative and conceptual synthesis rather than a systematic review or meta-analysis. Source selection was guided by conceptual relevance rather than exhaustive eligibility-based inclusion, and no formal methodological quality, risk-of-bias, or certainty-of-evidence assessment was performed. Consequently, the evidence synthesized here varies substantially in study design, participant characteristics, exercise modality and dose, endpoint definition, measurement approach, follow-up duration, and methodological rigor. The maturity of the evidence is also uneven across mechanistic domains: several cerebrovascular and peripheral biomarker endpoints have direct human evidence, whereas some blood–brain barrier-related, myokine, and circulating-factor pathways remain supported substantially by translational, disease-specific, or preclinical studies. This creates important limits for cross-species and mechanistic inference. Biological responses observed in experimental or disease-specific models may differ from those in aging humans in magnitude, timing, tissue origin, and functional relevance, while circulating concentrations of candidate biomarkers cannot be assumed to represent central nervous system activity. Differences in exercise dose, sampling timing, biological matrix, assay procedures, and participant characteristics may further contribute to apparently conflicting findings across studies. Biomarkers supported predominantly by translational or preclinical evidence should therefore be interpreted as hypothesis-generating supportive endpoints rather than established mediators of exercise-related cognitive effects until their temporal relationships, reproducibility, and human relevance are demonstrated more directly. The framework consequently reflects an integrative interpretation of heterogeneous evidence rather than a formal ranking of evidence strength or demonstration of causal mediation.

Second, publication and selection bias cannot be excluded. The published literature may preferentially represent positive or mechanistically coherent findings, whereas null or inconsistent biological responses may be less visible. In addition, because source prioritization in a narrative review necessarily involves author judgment, studies that aligned more closely with the mechanistic questions of this review may have been preferentially represented despite the structured search approach. These limitations should be considered when interpreting the relative prominence of individual pathways or endpoint domains within the synthesis.

Finally, the proposed framework remains conceptual and has not yet been prospectively validated as a study-design or interpretive tool. Its utility should therefore be regarded as a testable proposition rather than an established advantage. Future exercise–cognition studies could evaluate whether framework-informed designs improve the prespecification and alignment of participant vulnerability, delivered exercise exposure, mechanistic hypotheses, biological endpoints, and cognitive outcomes; whether they facilitate interpretation of null or heterogeneous responses and identification of plausible mediating or moderating factors; and whether they improve consistency and reproducibility across studies. Such evaluation should also consider feasibility, participant and measurement burden, resource requirements, and applicability across different exercise modalities, populations, and research settings. Prospective evaluation of these dimensions will be necessary before the framework can be considered a validated research tool or used to support clinical decision-making.

## 10. Conclusions

Exercise–cognition studies in aging are difficult to interpret when cognitive outcomes are considered without the delivered exercise stimulus, participant vulnerability, and intermediate biological responses. This review proposes a five-layer framework that aligns participant characteristics and exercise exposure with cerebrovascular mechanistic anchors, hypothesis-driven supportive biological endpoints, and cognitive or translational outcomes. Rather than prescribing a fixed endpoint battery, the framework provides an interpretive structure for studies seeking to clarify for whom, under what exercise conditions, and through which biological pathways exercise may support cognitive resilience in aging.

## Figures and Tables

**Table 1:** Cerebrovascular and supportive biological endpoint domains for mechanism-informed exercise–cognition studies.

Endpoint Domain	Examples of Measures	Mechanistic Relevance	Predominant Evidence Base	Interpretive Value	Main Limitations	Supporting Key References
Cerebral blood flow	Global or regional cerebral blood flow assessed by MRI, arterial spin labeling, transcranial Doppler, or related perfusion approaches	Reflects cerebral perfusion and oxygen/nutrient delivery, which may be relevant to vascular cognitive vulnerability	Direct human physiological and exercise evidence	Helps determine whether exercise is associated with perfusion-related adaptation	Resting blood flow does not necessarily indicate improved vascular reserve, cerebrovascular reactivity, or neurovascular coupling	[29,30,31,32,33,34]
Cerebrovascular reactivity	CO_2_ challenge, breath-hold response, MRI-based CVR, transcranial Doppler-based reactivity	Reflects the capacity of cerebral vessels to respond to vasoactive stimuli	Direct human physiological evidence; exercise evidence developing	Provides a more dynamic index of vascular responsiveness than resting perfusion alone	Results depend on stimulus type, gas-delivery method, imaging or recording modality, and analytic approach	[29,35,36]
Neurovascular coupling	Task-evoked BOLD fMRI, NIRS responses, or other measures linking neural activation and local vascular response	Captures coordination between neural activity, metabolic demand, and local vascular regulation	Direct human physiological evidence; exercise-specific evidence more limited	Useful when the hypothesis concerns functional brain activation and vascular responsiveness	Signals may reflect both neural and vascular factors; interpretation is difficult when vascular function differs across age or risk groups	[37,39]
Endothelial function	Flow-mediated dilation, peripheral arterial tonometry, circulating endothelial markers	Reflects systemic vascular health and nitric oxide-related vascular regulation	Substantial human vascular/exercise evidence; cerebral inference indirect when peripheral measures are used	May provide supportive evidence that exercise engages vascular pathways	Peripheral endothelial function does not automatically demonstrate improved cerebral vascular regulation	[2,8,29]
Arterial stiffness and vascular compliance	Pulse wave velocity, augmentation index, central blood pressure, vascular compliance indices	Reflects large-vessel aging, pulsatile flow transmission, and downstream microvascular stress	Substantial human vascular evidence	Relevant to vascular cognitive risk and long-term cerebrovascular burden	Associations with cognition do not establish direct exercise-induced brain vascular adaptation	[30,38]
Peripheral inflammatory markers	C-reactive protein, IL-6, TNF-α, IL-10, other cytokines or inflammatory profiles	Reflect systemic inflammatory status that may interact with vascular and cognitive vulnerability	Human observational and exercise-intervention evidence	May help identify broader biological responsiveness to exercise	Peripheral markers should not be overinterpreted as direct evidence of central neuroimmune change	[40,41,42]
Blood–brain barrier-related indicators	BBB permeability imaging, albumin ratio, vascular integrity markers, endothelial or tight-junction-related indicators	Relevant to vascular integrity, inflammatory regulation, and neurovascular health	Limited/heterogeneous human evidence; substantial translational and disease-specific evidence	May be informative in populations with vascular or metabolic vulnerability	Human evidence is heterogeneous; many mechanistic claims remain translational or disease-specific	[13,43]
Myokines and muscle-derived signals	BDNF, irisin/FNDC5-related markers, cathepsin B, GPLD1, clusterin, or other exercise-responsive circulating factors	May connect skeletal muscle contraction, metabolism, neuroplasticity, and brain health	Mixed evidence: Human data for selected factors, with substantial translational/preclinical support for proposed signaling pathways	Useful when interventions are expected to produce neuromuscular or metabolic adaptation	Circulating levels may not reflect central nervous system activity or causal mediation	[12,14,44,45,46,47,48]
Neurotrophic markers	BDNF and related neurotrophic indicators	Relevant to synaptic plasticity, neurogenesis-related hypotheses, and exercise-brain signaling	Human exercise evidence for circulating responses; causal mediation remains unestablished	May support interpretation of neuroplastic or systemic-to-brain signaling pathways	Responses vary by assay, timing, modality, training status, and participant characteristics	[40,44,45,46]
Cognitive outcomes	Executive function, processing speed, memory, attention, global cognition, functional cognition	Indicate clinical or functional relevance of exercise-related change	Direct human clinical and intervention evidence	Essential for determining whether biological adaptation is linked to meaningful cognitive outcomes	Practice effects, ceiling effects, outcome heterogeneity, and short follow-up may limit mechanistic interpretation	[4,22,23,24,25]

Note. BBB, blood–brain barrier; BDNF, brain-derived neurotrophic factor; BOLD, blood oxygen level-dependent; CO_2_, carbon dioxide; CVR, cerebrovascular reactivity; IL, interleukin; MRI, magnetic resonance imaging; NIRS, near-infrared spectroscopy; TNF-α, tumor necrosis factor-alpha.

**Table 2:** Exercise Modalities as Biological Stimuli for Mechanism-Informed Cognitive Aging Studies.

Exercise Modality	Primary Stimulus Profile	Mechanistic Pathways Most Plausibly Engaged	Endpoint Alignment	Interpretation Cautions	Supporting Key References
Aerobic exercise	Repeated cardiorespiratory and hemodynamic demand through walking, cycling, treadmill exercise, or other rhythmic endurance activities	Cardiorespiratory fitness, shear stress, endothelial function, cerebral perfusion, cerebrovascular reactivity, autonomic regulation, metabolic regulation	Cerebral blood flow, cerebrovascular reactivity, endothelial function, cardiorespiratory fitness, blood pressure, executive function, processing speed	Benefits should not be assumed without adequate intensity, progression, adherence, and delivered dose; vascular and cognitive responses may not follow the same time course	[8,9,10,32,33,34,49,50]
Resistance training	Repeated neuromuscular loading through machine-based, free-weight, elastic-band, or body-weight exercise	Muscle strength, insulin sensitivity, functional capacity, muscle-derived signaling, neuromuscular reserve, metabolic health	Strength, functional performance, metabolic markers, myokines, neurotrophic markers, selected cognitive domains such as executive function	Should not be interpreted as superior or inferior to aerobic exercise; mechanistic relevance depends on whether the study hypothesis involves neuromuscular, metabolic, or muscle–brain pathways	[12,14,50,51,52]
Multicomponent exercise	Combined aerobic, resistance, balance, coordination, flexibility, or functional training components	Broader physical function, mobility, balance, cardiorespiratory and neuromuscular adaptation, cognitive engagement, adherence potential	Functional capacity, mobility, balance, fitness, strength, cognitive outcomes, feasibility, adherence, selected vascular or metabolic endpoints	Component effects may be difficult to separate; reporting must specify component content, progression, intensity, and delivered dose	[50,53,54]
Mind–body exercise	Coordinated movement, posture control, breathing, attention, and often social or group-based participation, as in Tai Chi or Qigong	Balance, coordination, attentional engagement, autonomic regulation, stress modulation, adherence, social participation	Balance, mobility, executive function, attention, adherence, quality of participation, autonomic or stress-related indicators where relevant	Should not be assumed to produce the same hemodynamic or vascular stimulus as moderate-to-vigorous aerobic training	[55]
Low-intensity functional movement	Light walking, mobility exercise, stretching, functional movement practice, or low-load activities adapted to frailty or low tolerance	Movement confidence, functional maintenance, sedentary interruption, safety, gradual conditioning	Feasibility, tolerance, physical function, sedentary behavior, safety, patient-reported outcomes	May be highly relevant for vulnerable participants, but mechanistic expectations should match the likely stimulus intensity	[49]
Combined exercise plus behavioral support	Exercise combined with supervision, coaching, self-monitoring, social support, or strategies intended to improve intervention delivery	Delivered dose, adherence, maintenance, self-efficacy, behavioral engagement, long-term feasibility	Adherence, retention, delivered dose, feasibility, maintenance, cognitive and functional outcomes	Behavioral support may influence outcomes independently of physiological exercise dose; interpretation should separate stimulus delivery from behavioral context	[15,16,49]
Higher-intensity or progressive training	Structured progression toward higher aerobic or resistance intensity when tolerated	Stronger cardiorespiratory, vascular, metabolic, or neuromuscular stimulus	Fitness, strength, vascular responsiveness, metabolic markers, adverse events, recovery, tolerance	May increase biological stimulus but also increase burden; tolerance and safety must be interpreted in relation to baseline vulnerability	[17,49]

Note. This table is intended to support mechanistic interpretation rather than to rank exercise modalities or provide clinical prescription guidance. The appropriate modality depends on the hypothesis, participant vulnerability, delivered dose, and selected endpoints.

**Table 3:** Mechanism-Informed Interpretation Matrix for Future Exercise–Cognition Studies.

Design or Interpretation Question	Mechanism-Informed Consideration	Endpoint Implication	Main Caution	Supporting Key References
What biological pathway is the exercise intervention expected to engage?	The intervention should be linked to a plausible vascular, metabolic, neuromuscular, inflammatory, neurotrophic, or behavioral mechanism	Select endpoints that correspond to the expected pathway rather than adding broad outcome panels	A cognitive change alone does not identify the biological pathway involved	[8,12,13,14,29,30,31,32,33,34,35,36,37,38,39,40,41,42,43,44,45,46,47,48]
Is the prescribed exercise stimulus adequately described?	Modality, frequency, intensity, duration, progression, supervision, and adherence should be reported clearly	Exercise exposure should be interpretable as a biological stimulus, not only as a behavioral label	Poor intervention reporting limits replication and weakens mechanistic interpretation	[15,16,17,49]
Was the delivered dose sufficient and tolerable for the study population?	Baseline fitness, frailty, vascular risk, comorbidity, fatigue, and recovery capacity may influence whether the intended stimulus is achieved	Delivered dose, adherence, tolerance, and progression should be considered alongside biological and cognitive outcomes	A null cognitive effect may reflect insufficient or poorly tolerated exposure rather than biological inactivity	[18,28,49,56,57,58,59,60,61]
Are cerebrovascular endpoints aligned with the hypothesis?	Cerebral blood flow, cerebrovascular reactivity, endothelial function, vascular compliance, and neurovascular coupling reflect different aspects of vascular function	The selected cerebrovascular endpoint should match the pathway being tested	Cerebrovascular measures are not interchangeable and require method-specific interpretation	[29,30,31,32,33,34,35,36,37,38,39]
Are supportive biological markers justified?	Inflammatory markers, blood–brain barrier-related indicators, myokines, and neurotrophic factors may clarify broader biological responsiveness	Supportive markers should be selected according to modality, population vulnerability, and expected adaptation	Peripheral biomarkers should not be overinterpreted as direct evidence of central neuroimmune change	[13,40,41,42,43,44,45,46,47,48]
Is the cognitive outcome sensitive to the expected mechanism?	Cognitive domains differ in their relevance to vascular, neural, metabolic, and functional pathways	For vascular-centered hypotheses, prioritize domain-specific measures of executive function and processing speed; select other domains according to the proposed mechanism. Use validated alternate forms and standardized retest conditions where feasible	Practice effects, ceiling effects, baseline cognitive status, test heterogeneity, and reliance on global screening measures may obscure mechanistic interpretation; no cognitive domain is uniquely specific to one biological pathway	[4,22,23,24,25,56,57,58,59]
How should participant vulnerability be handled?	Vascular risk, metabolic burden, cognitive status, sex, fitness, medication use, and comorbidity may modify response	Baseline vulnerability should be considered prospectively in design and analysis	Vulnerability should not be used only as a post hoc explanation for inconsistent findings	[1,2,3,19,56,57,58,59]
How should responder heterogeneity be interpreted?	Apparent response differences may reflect true biological variability, adherence, baseline status, measurement error, or statistical classification	Responder analyses should be linked to exercise exposure and mechanistic endpoints	Simple responder/non-responder labels may be misleading without measurement and statistical caution	[18,28,60,61]
How should relationships among framework layers be analyzed?	Repeated outcomes can be examined longitudinally, baseline vulnerability can be tested as a moderator, and candidate biological pathways can be evaluated as mediators when temporal ordering is available	Mixed-effects models can evaluate trajectories; prespecified interaction terms can test moderation; longitudinal mediation models can examine indirect pathways linking exercise exposure, biological change, and cognitive outcomes	Statistical mediation does not establish biological causality; moderator tests should be prespecified where possible, and post hoc responder classification should be avoided	[18,28,62,63,64]
What makes the study mechanistically interpretable?	Strong interpretation requires alignment among participant vulnerability, exercise stimulus, biological endpoints, and cognitive or translational outcomes	The endpoint battery should be hypothesis-driven and proportionate to the study aim	Measuring more endpoints does not necessarily improve interpretation if their roles are unclear	[15,16,17,62,63,64]

Note. This matrix is not a formal guideline or endpoint checklist. It is intended to help align intervention design, endpoint selection, and interpretation in future exercise–cognition studies.

## Data Availability

No new data were created or analyzed in this study. Data sharing is not applicable to this article.

## Abbreviations

The following abbreviations are used in this manuscript:

ASL	Arterial spin labeling
BBB	Blood–brain barrier
BDNF	Brain-derived neurotrophic factor
BOLD	Blood oxygen level-dependent
CERT	Consensus on Exercise Reporting Template
CO_2_	Carbon dioxide
CVR	Cerebrovascular reactivity
fMRI	Functional magnetic resonance imaging
FNDC5	Fibronectin type III domain-containing protein 5
GPLD1	Glycosylphosphatidylinositol-specific phospholipase D1
IL	Interleukin
IL-6	Interleukin-6
IL-10	Interleukin-10
MRI	Magnetic resonance imaging
NIRS	Near-infrared spectroscopy
PGC-1α	Peroxisome proliferator-activated receptor gamma coactivator 1-alpha
TCD	Transcranial Doppler
TIDieR	Template for Intervention Description and Replication
TNF-α	Tumor necrosis factor-alpha

## Appendix A

Table A1 provides a pragmatic summary of representative procedures and timing considerations for selected endpoints emphasized in the framework. The suggested timing reflects the distinction between acute exercise responses and chronic training adaptations and should be prespecified according to the study hypothesis rather than interpreted as a mandatory protocol.

**Table A1:** Practical measurement procedures and timing considerations for selected mechanistic endpoints in exercise–cognition studies.

Endpoint/Method	Representative Procedure	Primary Information Obtained	Typical Timing Relative to Exercise	Relative Feasibility	Key Interpretive Considerations	Supporting References
Cerebral perfusion—ASL MRI	Resting arterial spin labeling MRI with standardized acquisition and physiological conditions	Quantitative or regional cerebral perfusion	Chronic studies: baseline and post-intervention under comparable resting conditions; post-training assessment should use a prespecified interval from the final exercise session when chronic adaptation is the target. Acute studies: pre-exercise and predefined post-exercise imaging when acute perfusion change is the hypothesis	Moderate to low; requires MRI access and standardized acquisition/analysis	Sensitive to physiological state and acquisition protocol; resting perfusion should not be interpreted as equivalent to CVR or neurovascular coupling	[29,30,31,32,33,34]
CVR—MRI with CO_2_ or related vasoactive challenge	BOLD- or perfusion-based MRI during controlled CO_2_ exposure or another standardized vasoactive stimulus	Cerebrovascular responsiveness or vascular reserve	Chronic studies: baseline and post-intervention using the same stimulus, acquisition, and physiological conditions, with timing relative to the last session standardized. Acute studies: pre-exercise and predefined post-exercise assessment if acute vascular responsiveness is of interest	Low; requires MRI and stimulus-control infrastructure	Results depend on stimulus delivery, imaging sequence, physiological monitoring, and analytic approach; protocols should remain consistent across visits	[35,36]
CVR—TCD with CO_2_ or breath-hold challenge	Middle cerebral artery blood-flow velocity recorded by TCD during standardized hypercapnic or breath-hold challenge	Dynamic cerebrovascular responsiveness based on changes in blood-flow velocity	Chronic studies: baseline and post-intervention under matched resting conditions. Acute studies: pre-exercise and early post-exercise or serial post-exercise assessments according to the hypothesis	High to moderate; portable and comparatively accessible	Measures velocity rather than absolute regional cerebral blood flow; interpretation assumes stable insonated-vessel characteristics and requires consistent probe position and stimulus delivery	[29,35,36]
TCD—resting or exercise-related cerebral blood-flow velocity	Continuous or repeated TCD recording of cerebral blood-flow velocity at rest, during exercise, or recovery	Temporal changes in cerebral hemodynamics	Acute studies: baseline, during exercise when feasible, and predefined recovery periods. Chronic studies: standardized resting measurements before and after the intervention	High to moderate	Useful for temporal resolution but does not provide regional perfusion imaging; carbon dioxide, blood pressure, posture, and exercise intensity may influence responses	[29,30,31,32,33,34]
Neurovascular coupling—task-based BOLD fMRI or NIRS	Standardized cognitive or sensorimotor task paired with BOLD fMRI, NIRS, or related hemodynamic recording	Coupling between task-related neural demand and vascular response	Baseline and post-intervention using the same task and acquisition protocol; acute designs require clearly defined pre/post windows	MRI: low; NIRS: moderate to high	Signal reflects interacting neural and vascular components; task familiarity and vascular differences across age or risk groups may affect interpretation	[37,39]
Circulating BDNF	Serum or plasma collection using the same biological matrix, collection procedure, processing protocol, and assay across visits	Peripheral neurotrophic response associated with exercise and neuroplasticity-related hypotheses	Acute studies: pre-exercise and predefined immediate/early post-exercise sampling, with additional samples when response kinetics are of interest. Chronic studies: resting baseline and post-intervention sampling under matched conditions and standardized timing relative to the final exercise session	High; requires blood collection and laboratory assay	Serum and plasma values are not interchangeable; exercise timing, assay procedure, training status, and participant characteristics may influence measured concentrations; peripheral BDNF does not directly represent central nervous system activity	[40,44,45,46]

Note. ASL, arterial spin labeling; BDNF, brain-derived neurotrophic factor; BOLD, blood oxygen level-dependent; CO_2_, carbon dioxide; CVR, cerebrovascular reactivity; MRI, magnetic resonance imaging; NIRS, near-infrared spectroscopy; TCD, transcranial Doppler. Timing recommendations are intended as pragmatic design considerations rather than universal protocol requirements. Acute and chronic responses should be distinguished prospectively, and repeated measurements should use consistent physiological conditions, acquisition procedures, and timing relative to exercise whenever possible.
